# Efgartigimod combined with rituximab helps improve the symptoms and reduce the use of corticosteroids in patients with MuSK antibody-positive MG: a single case report

**DOI:** 10.3389/fimmu.2025.1710017

**Published:** 2026-01-07

**Authors:** Xiujun Zheng, Jing Wang, Tongtong Cai, Houshi Zhou

**Affiliations:** 1 Department of Neurology, Shantou Central Hospital, Shantou, China; 2 Department of Neurology, The First Affiliated Hospital of Sun Yat-sen University, Guangzhou, China

**Keywords:** myasthenia gravis (MG), MuSK, efgartigimod, rituximab, case report

## Abstract

**Introduction:**

Myasthenia gravis (MG) is an autoimmune disorder primarily affecting the neuromuscular junction. Muscle-specific receptor tyrosine kinase antibody (MuSK-Ab)-mediated MG often presents with bulbar weakness and is prone to crisis. Currently, conventional treatments show limited efficacy in MuSK-Ab-mediated MG, while rituximab therapy demonstrates favorable outcomes.

**Case presentation:**

We report a case of a patient who initially achieved symptom control with efgartigimod during an acute exacerbation but experienced symptom recurrence after subsequent rituximab treatment. Owing to poor tolerance to pyridostigmine and corticosteroids, and considering the established efficacy of efgartigimod in MuSK-Ab-positive MG, we opted for continued efgartigimod infusions. This approach quickly improved symptoms, achieved minimal symptom expression (MSE), and allowed faster tapering of corticosteroids and pyridostigmine.

**Conclusion:**

While multiple studies have demonstrated the efficacy and safety of efgartigimod, large-scale studies remain necessary to further evaluate the feasibility of combination therapy with rituximab in MuSK-Ab-positive MG.

## Introduction

Myasthenia gravis (MG) is an autoimmune disorder primarily caused by antibodies against the acetylcholine receptor (AChR) that interfere with the transmission of nerve–muscle connections. Additionally, antibodies targeting muscle-specific receptor tyrosine kinase (MuSK), low-density lipoprotein receptor-related protein 4 (LRP4), or agrin can also lead to the disease. The MuSK antibodies (MuSK-Ab) (approximately 5%–8%) belong to the immunoglobulin G4 (IgG4) type and fail to activate complement, the titer of which is related to the severity of the disease. More than 40% of patients with MuSK-MG present with bulbar weakness, usually accompanied by cervical and respiratory involvement. Compared with other subgroups of MG, patients with MuSK-Ab exhibit a poorer response to cholinesterase inhibitors and depend more on steroids while responding better with rituximab, which contributes to more tolerance for the reduction in oral immunosuppressants and corticosteroids (1–5). Therefore, rituximab can be given priority when initial immunotherapy is ineffective for patients with MuSK-MG.

Efgartigimod derived from the human immunoglobulin G1 (IgG1) Fc domain binds to endogenous IgG neonatal Fc receptor (FcRn) and reduces the titer of pathogenic IgG antibodies. At present, the phase 3 clinical trial of efgartigimod in generalized MG has been completed, demonstrating long-term safety, tolerability, and efficacy. Patients who used efgartigimod saw a reduction in illness load, an increase in strength, and a higher quality of life (6). Multiple studies have reported that the therapeutic effect is both sustainable and repeatable (7, 8). Additionally, efgartigimod treatment for MuSK-MG has been shown to improve clinical symptoms and reduce steroid dosage (9).

The diagnosis and treatment of MuSK-MG are challenging, which is embodied in its rarity, reliance on steroids, and the high risk of myasthenia crises (7). Consequently, further clinical experience in the management of MuSK-MG is warranted. In this report, we present a case of MG exhibiting marked intolerance to both corticosteroids and cholinesterase inhibitors. On the basis of treatment with rituximab, he received periodic infusions of efgartigimod that helped the patient quickly reach the minimal symptom expression (MSE) and facilitated the rapid reduction of hormones and pyridostigmine.

## Case presentation

A 54-year-old male patient developed diplopia in 2023 without an identifiable precipitating factor. In October 2024, he was admitted to the hospital due to the acute onset of generalized weakness, dysphagia, dysarthria, and dyspnea. Within 10 days, he experienced two episodes of unconsciousness with unresponsiveness to verbal stimuli and received endotracheal intubation with mechanical ventilation, antibiotic therapy, volume expansion, and anti-inflammatory treatment (glucocorticoids). Electromyography (EMG) indicated a decrement in compound muscle action potential (CMAP) amplitudes of the ulnar and accessory nerves under low-frequency (3 Hz) repetitive nerve stimulation. Serological test was positive for MuSK-Ab (1.19 nmol/L, reference value ≤0.05 nmol/L) by radioimmunoassay. Consequently, the patient was diagnosed with “myasthenia gravis”. Thyroid function tests and the thymus computed tomography examination were normal, and the autoimmune antibody panels, including antinuclear antibody (ANA) and antineutrophil cytoplasmic antibody (ANCA), were negative.

Following a confirmed diagnosis, the patient was treated with efgartigimod (10 mg/kg weekly for 4 weeks) to facilitate rapid recovery during the acute exacerbation phase. Concurrently, the patient received pyridostigmine bromide (60 mg three times daily) and prednisone with the dose gradually increased to 30 mg/day. During this period, the patient showed significant improvement in limb and neck weakness, as well as respiratory symptoms, which allowed for successful extubation, weaning from mechanical ventilation, and transfer to a general ward. However, symptoms such as dysphagia persisted and the MG-specific activities of daily living (ADL) score is 7 and the quantitative myasthenia gravis (QMG) score is 9. Meanwhile, the patient experienced adverse effects attributed to pyridostigmine bromide and prednisone, including abdominal pain and significant weight gain (20 kg within 2 months). After completion of the efgartigimod treatment cycle, the patient received rituximab 500 mg to target B lymphocytes. Following this, the patient’s swallowing function improved markedly, permitting removal of the nasogastric tube (scores: OMG: 7; ADL: 6). Monitoring revealed that the B-lymphocyte percentage had decreased to 0 (compared to a pre-treatment level of 0.41×10^9^/L.

Five days later, the patient experienced recurrence of diplopia, dysphagia, and neck weakness due to fatigue and sleep disorders (scores: OMG: 20; ADL: 15). He continued to receive another cycle of treatment with efgartigimod. The daily dose of prednisone was increased by 5 mg at weekly intervals up to 50 mg/day. Symptoms showed mild improvement following this (scores: MG-ADL: 9, QMG: 13). After 2 months of efgartigimod use, the dosing interval was extended to every 2 weeks and prednisone was maintained at 50 mg daily. At this moment, the patient also reached the MSE state (scores: MG-ADL: 0, QMG: 1) and maintained for 8 weeks.

**Figure 1:**
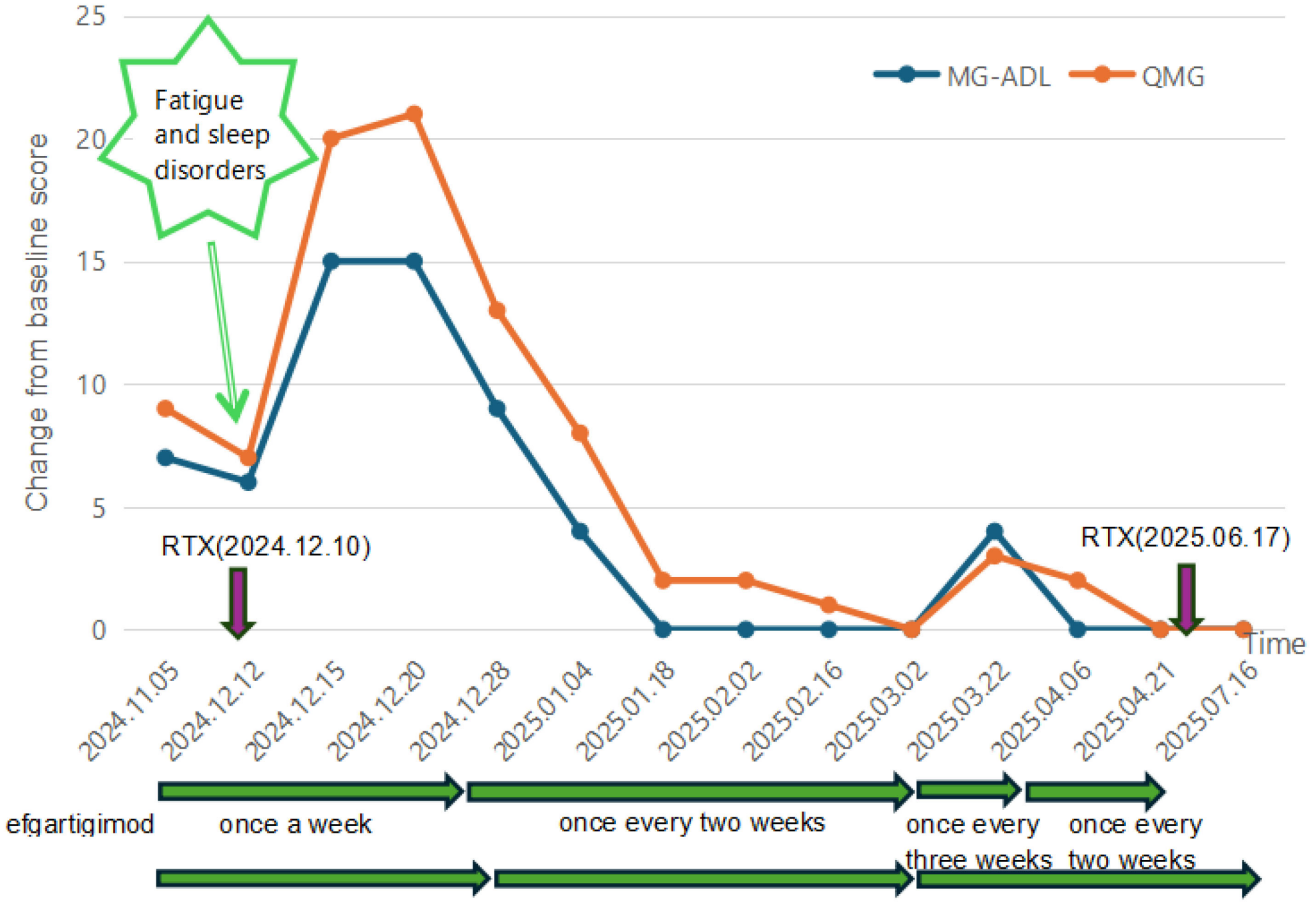
The line graph shows the changes in MG-ADL and QMG scores during the patient’s medication process. The purple arrow indicates the time when rituximab was used, the green arrow represents the interval during which Efgartigimod was administered, and the red arrow indicates the process of adjusting the dose of prednisone. RTX, rituximab. The patient received the first cycle of Efgartigimod at another hospital during the acute exacerbation period, and no specific scoring data were available during this period. The intervals between each injection of Efgartigimod in the figure are all the times as planned. However, the actual injection times may have slight differences from the estimated times.

Because of concerns regarding the possible side effects of corticosteroids and pyridostigmine (the abdominal pain and the weight gain), during the second month after discharge, the patient continued efgartigimod as an adjunct treatment to facilitate reduction in the dosages of pyridostigmine and prednisone. The dosage of pyridostigmine was reduced to 120 mg/day, and the daily dose of prednisone was reduced by 5 mg each week. Motivated by financial considerations, the patient further extended the interval between efgartigimod doses during the fifth cycle but symptoms (diplopia, etc.) recurred 2 weeks and 5 days after the last dose (scores: MG-ADL: 4, QMG: 3). Consequently, the physician recommended increasing pyridostigmine to 180 mg/day, increasing prednisone to 20 mg/day, and continuing efgartigimod every 2 weeks. Symptoms gradually improved, and the patient achieved MSE status again 1 month later (scores: MG-ADL: 0, QMG: 0). The specific changes in the scores can be found in **Figure 1**.

**Figure 2:**
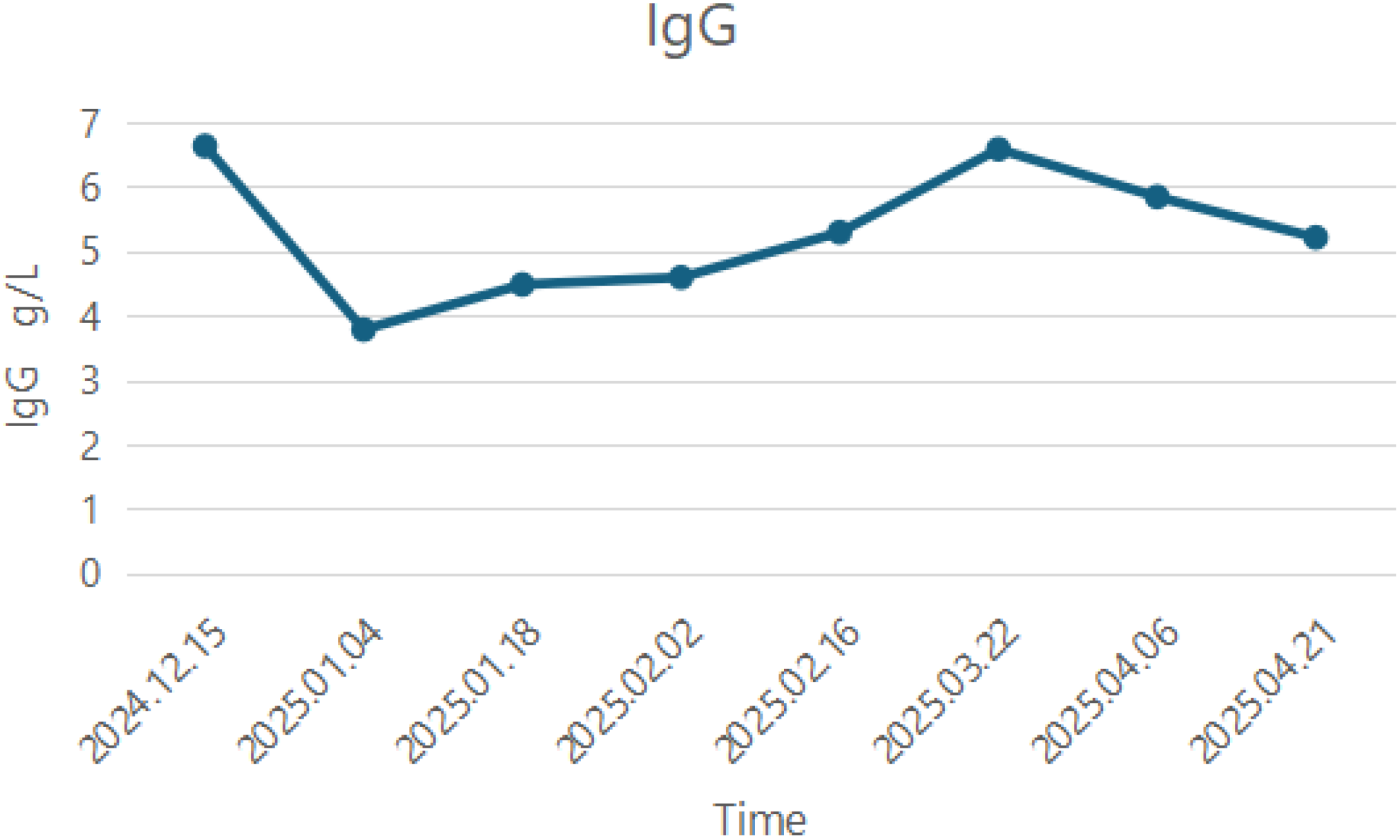
The changes in immunoglobulin G (IgG) levels during the use of Efgartigimod.

**Figure 3:**
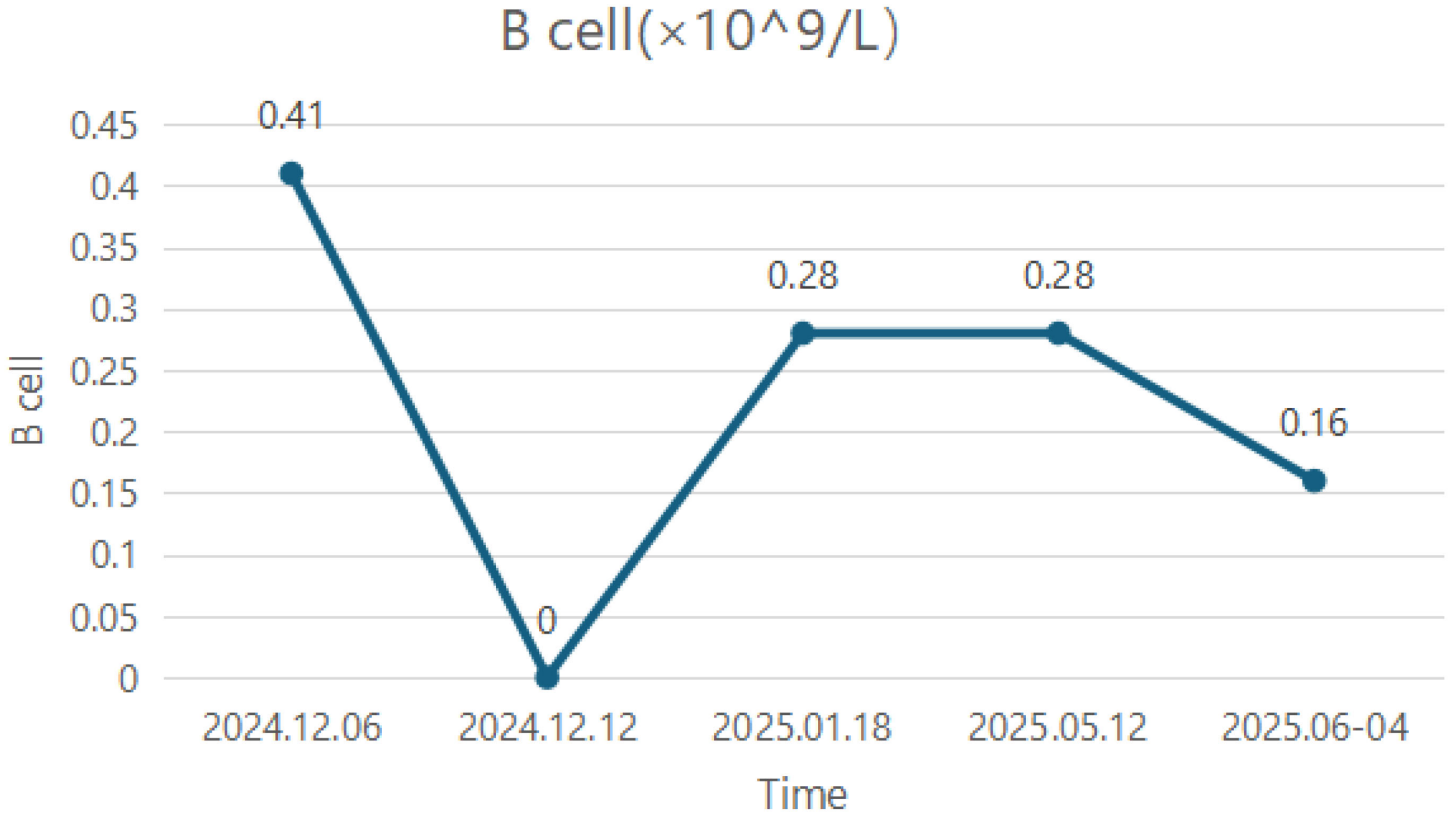
The changes in B-cell counts after the first infusion of rituximab.

The last time efgartigimod was used was on 12 May 2025, and a total of 4 cycles were administered. The detailed changes in IgG levels are presented in **Figure 2**. In May 2025, the patient’s B-lymphocyte count was 0.28×10^9^/L and the detailed numerical values of B-cell changes are presented in **Figure 3**. To further reduce the B-lymphocyte levels and based on the guideline recommendations (8), the patient received a second cycle of rituximab 500 mg, administered approximately 6 months after the first dose. As of 12 July 2025, the patient’s symptoms stably remained MSE.

## Discussion

We describe a 54-year-old man with MuSK-Ab positive who regularly used efgartigimod in combination with rituximab. This treatment helped the patient overcome myasthenic crises (MCs), improve symptoms, maintain an MSE state, and assist in the rapid reduction of pyridostigmine and prednisone.

MuSK is a transmembrane tyrosine kinase critical for postsynaptic clustering of AChRs. MuSK-Ab disrupts MuSK function, leading to AChR cluster disintegration and neuromuscular junction failure (4). Rituximab, a monoclonal antibody targeting CD20 on B cells, induces circulating B-cell depletion and rapidly reduces pathogenic antibody titers, which facilitates minimal disease manifestations, improves prognosis, and reduces reliance on other immunosuppressants (5, 9, 10). Its efficacy is particularly pronounced in MuSK-MG due to high CD20 expression on circulating plasmablasts producing MuSK antibodies (11). However, the lack of CD20 expression on long-lived plasma cells—the primary source of circulating immunoglobulins—explains the slow decline in antibody titers and the consequent delayed onset of action (12).

The neonatal Fc receptor (FcRn) binds IgG in acidic endosomal compartments and releases it at physiological pH, circumventing lysosomal degradation and prolonging IgG half-life (13). Efgartigimod, an FcRn antagonist with enhanced affinity and pH-dependent binding, reduces IgG recycling and accelerates degradation of pathogenic IgG antibodies (6). Preclinical studies show that intraperitoneal efgartigimod in MuSK-MG mice reduced IgG4 titers by eightfold (14). Moreover, efgartigimod demonstrated a safe and sustainable reduction in human IgG4 levels, achieving a maximum percentage reduction ranging from 36.6% to 46.0%, which was marginally lower than that observed for IgG1–IgG3 (15). Clinical evidence supports efgartigimod’s rapid control of GMG exacerbations (MGAE) and MC, enabling symptom improvement and accelerated steroid tapering in MuSK-MG (6, 16). Real-world data further indicate its utility in long-term GMG management (17). Moreover, the advantages include cyclic administration flexibility and no requirement for vaccination (18).

Considering this patient’s specific needs, we are using a combination of long-acting rituximab (B-cell depletion at 3–6 months (19)) and short-acting efgartigimod (half-life of 80–120 h (15)). Rituximab ensures that B cells producing antibodies remain at a low level, while efgartigimod controls the titer of pathogenic IgG4 antibodies within each shorter treatment cycle. This dual therapy targets both the upstream and downstream mechanisms of the disease pathogenesis, thus promoting rapid achievement of MSE status and enabling rapid steroid tapering.

It is noteworthy that in our patient, the B-lymphocyte count rebounded to 0.28×10^9^/L 1 month after rituximab administration. The potential reasons for this include the following:

1. CD20 is primarily expressed on mature B lymphocytes but shows lower expression on precursor B cells, plasmablasts, and plasma cells, which the B-cell depletion therapy cannot completely eliminate (12).
2. Rituximab mainly targets circulating B cells, while tissue-resident B cells are more difficult to eradicate and may undergo homing phenomena (20).
3. A second cycle of efgartigimod was initiated several days after rituximab administration. Rituximab itself is an IgG1 monoclonal antibody, and efgartigimod, while clearing pathogenic antibodies, may also reduce the titers of beneficial antibodies (15), thereby potentially diminishing the therapeutic effect of rituximab.

This patient was initially planned to receive only one cycle of efgartigimod to rapidly overcome the crisis period, followed by rituximab several days later according to guidelines (8). However, after rituximab administration, the patient experienced a worsening of MG symptoms due to factors such as fatigue and poor sleep. To achieve rapid symptom control, stabilization, and accelerated steroid tapering, multiple cycles of efgartigimod were reintroduced. The second course of rituximab was initiated 1 month after discontinuing efgartigimod, aiming to space out the treatments sufficiently to minimize potential drug–drug interactions.

Mechanistically, efgartigimod and rituximab should not be used in combination. However, given this patient’s symptom fluctuations and specific clinical needs, we opted for this personalized treatment approach. Nevertheless, the optimal interval between efgartigimod and rituximab administration to minimize mutual interference remains unknown and requires further investigation with larger patient datasets.

Retrospective studies report MG-ADL score reductions from 12.00 ± 5.48 to 5.75 ± 4.79 after one efgartigimod cycle (21), and from 10.1 ± 4.0 to 2.2 ± 3.1 when combined with maintenance immunosuppressants (22). A case study documented MG-ADL improvement from 9 to 0 and QMG from 20 to 8 after a cycle of efgartigimod in MuSK-MG exacerbation (23). In contrast, our patient achieved a 15-point MG-ADL and an 18-point QMG reduction after two efgartigimod cycles plus rituximab, attaining MSE. However, this may be due to patients with higher baseline scores having greater potential for clinical improvement following treatment. Stable symptoms permitted the daily dose of prednisone tapering at 5 mg/week—faster than the conventional 5-mg biweekly reduction after 6–8 weeks of stability, which is attributed to the significant role played by efgartigimod.

Standard efgartigimod dosing is 10 mg/kg weekly per 4-week cycle. However, its efficacy and duration vary among patients with MuSK-MG, necessitating individualized regimens (6, 17, 24). Owing to economic constraints, our patient extended efgartigimod dosing intervals to 2 weeks during subsequent cycles, resulting in elevated IgG levels (**Figure 2**). Because of concurrent prednisone adjustments, symptoms remained stable until an interval of 2 weeks + 5 days provoked symptom recurrence and IgG rebound, prompting reversion to 2-week dosing. This suggests that 2-week intervals represent an optimal balance between cost-effectiveness and disease control for this patient.

Efgartigimod significantly reduces the composite burden of myasthenic crises, exacerbations, hospitalizations, and rescue medication requirements. Rituximab monotherapy similarly reduces the need for rescue medications (9, 18). In our case, the patient required no intensive care unit (ICU) admissions, endotracheal intubation, or emergent interventions during the 6 months post-MG diagnosis. When exacerbations occurred, they were readily managed without escalation to crisis-level care—an outcome we attribute to the combined use of rituximab and efgartigimod. This suggests that dual therapy further mitigates the probability of myasthenic crises and rescue medication utilization.

## Conclusion

Collectively, the synergistic targeting of upstream (B-cell depletion via rituximab) and downstream (pathogenic IgG clearance via efgartigimod) pathogenic mechanisms in MG facilitates rapid symptomatic improvement accelerated tapering of corticosteroids and pyridostigmine reduced frequency of myasthenic crises and ICU admissions. Nevertheless, therapeutic regimens require rigorous individualization. Total efgartigimod treatment duration and the tapering velocity of prednisone must be tailored to disease severity, clinical response, and patient tolerance profiles. Furthermore, as a single case report, our findings are subject to reporting bias. Large sample cohort studies and retrospective analyses are warranted to validate the efficacy and safety of this combinatorial approach.
